# Proton Irradiation Increases the Necessity for Homologous Recombination Repair Along with the Indispensability of Non-Homologous End Joining

**DOI:** 10.3390/cells9040889

**Published:** 2020-04-05

**Authors:** Klaudia Szymonowicz, Adam Krysztofiak, Jansje van der Linden, Ajvar Kern, Simon Deycmar, Sebastian Oeck, Anthony Squire, Benjamin Koska, Julian Hlouschek, Melanie Vüllings, Christian Neander, Jens T. Siveke, Johann Matschke, Martin Pruschy, Beate Timmermann, Verena Jendrossek

**Affiliations:** 1 Institute of Cell Biology (Cancer Research), University Hospital Essen, University of Duisburg-Essen, 45147 Essen, Germany; klaudia.szymonowicz@uk-essen.de (K.S.); adam.krysztofiak@uk-essen.de (A.K.); janettevanderlinden@hotmail.com (J.v.d.L.); sebastian.oeck@uk-essen.de (S.O.); julian.hlouschek@uk-essen.de (J.H.); johann.matschke@uk-essen.de (J.M.); 2 West German Proton Therapy Centre Essen (WPE), West German Cancer Center (WTZ), University Hospital Essen, 45147 Essen, Germany; ajvar.kern@uk-essen.de (A.K.); benjamin.koska@uk-essen.de (B.K.); melanie.vuellings@uk-essen.de (M.V.); beate.timmermann@uk-essen.de (B.T.); 3 Department of Radiation Oncology, Laboratory for Applied Radiobiology, University Hospital Zurich, Zurich, Switzerland; simon.deycmar@uzh.ch (S.D.); martin.pruschy@uzh.ch (M.P.); 4 Department of Therapeutic Radiology, Yale University School of Medicine, New Haven, CT 06520, USA; 5 Institute of Experimental Immunology and Imaging, Imaging Center Essen, University Hospital Essen, 45122 Essen, Germany; anthony.squire@uk-essen.de; 6 Institute of Developmental Cancer Therapeutics, West German Cancer Center, University Hospital Essen, Essen, Germany; christian.neander@uk-essen.de (C.N.); jens.siveke@uk-essen.de (J.T.S.); 7 Division of Solid Tumor Translational Oncology, German Cancer Consortium (DKTK, partner site Essen) and German Cancer Research Center, DKFZ, 69120 Heidelberg, Germany; 8 Department of Particle Therapy, West German Proton Therapy Center Essen (WPE), West German Cancer Center (WTZ), University Hospital Essen, 45147 Essen, Germany

**Keywords:** ionizing radiation, DNA damage foci, DNA repair, spread-out Bragg peak (SOBP), relative biological effectiveness (RBE), entrance plateau (EP) protons, linear energy transfer (LET), DNA double-strand break (DSB), non-homologous end joining (NHEJ), homologous recombination repair (HRR), relative biological effectiveness (RBE)

## Abstract

Technical improvements in clinical radiotherapy for maximizing cytotoxicity to the tumor while limiting negative impact on co-irradiated healthy tissues include the increasing use of particle therapy (e.g., proton therapy) worldwide. Yet potential differences in the biology of DNA damage induction and repair between irradiation with X-ray photons and protons remain elusive. We compared the differences in DNA double strand break (DSB) repair and survival of cells compromised in non-homologous end joining (NHEJ), homologous recombination repair (HRR) or both, after irradiation with an equal dose of X-ray photons, entrance plateau (EP) protons, and mid spread-out Bragg peak (SOBP) protons. We used super-resolution microscopy to investigate potential differences in spatial distribution of DNA damage foci upon irradiation. While DNA damage foci were equally distributed throughout the nucleus after X-ray photon irradiation, we observed more clustered DNA damage foci upon proton irradiation. Furthermore, deficiency in essential NHEJ proteins delayed DNA repair kinetics and sensitized cells to both, X-ray photon and proton irradiation, whereas deficiency in HRR proteins sensitized cells only to proton irradiation. We assume that NHEJ is indispensable for processing DNA DSB independent of the irradiation source, whereas the importance of HRR rises with increasing energy of applied irradiation.

## 1. Introduction

Radiotherapy is a part of the standard treatment for more than 50% of cancer patients and has a documented contribution to local tumor control and improved overall survival. Clinical radiotherapy aims to achieve maximal tumor control rates while reducing the risk for dose-limiting adverse late effects in normal tissues [1,2]. Depending on the disease stage, radiotherapy is therefore either given alone or as a part of multimodal combinations with surgery, chemotherapy and molecular-targeted drug therapy or immunotherapy [1,3]. Overcoming dose-limiting toxicity to normal tissues is still a major challenge in clinical radiotherapy, particularly when tumors grow adjacent to critical structures or within tissues or organs with pronounced radiation sensitivity. Enhancing the accuracy of dose delivery to the tumor volume, e.g., by stereotactic radiotherapy or intensity-modulated radiation therapy, has allowed clinicians to improve the safety profile of radiotherapy for many solid tumors [4]. Moreover, recent technical developments have led to broader application of particle therapy in clinical practice. It is expected that the favorable depth-dose curves and linear energy transfer (LET) characteristics of charged particles will allow a precise targeting of deep-seated tumors while reducing the ionizing dose and irradiated volume of normal tissue. It is expected that these attempts to achieve a precise and more target-specific irradiation will help to lower the toxicity rates and the risk of developing secondary tumors [5,6,7,8,9,10,11]. In fact, particles pass through normal tissues on their track without losing much energy but instead releasing the main carried energy at a specific tissue depth shortly before their complete stop. This maximized dose deposition at the end of the particle’s range is represented as a Bragg peak of the depth-dose curve. In contrast, photons lose their energy exponentially, with higher values at the entry point and lower values at the deep-seated tissues [12,13,14]. Additionally, the rising number of particle therapy centers worldwide facilitates the use of protons or heavy ions in the clinical care of cancer patients and allows the validation of the suspected superiority of particle beams compared to photon beams in terms of normal tissue protection [15,16]. 

To allow for irradiation of a three-dimensional tumor volume, therapeutic proton beam therapy is performed using a so called spread-out Bragg peak (SOBP), which is composed of several single Bragg peaks with slightly varying energies [17]. Despite divergent physical properties of proton and photon beams, it is assumed that the relative biological effectiveness (RBE) of proton beams resemble those of photon beams, mostly when put in relation to high LET particle beams such as carbon ions [18]. As a consequence, treatment planning for proton beam radiotherapy has been developed based on data originating from therapy with gamma-ray photons generated by a ^60^Co source, using a correction factor for RBE of 1.1 [18,19,20,21,22]. Herein, RBE is defined as the ratio of biological effectiveness of two radiation modalities, measured by absorbed dose for a given effect (reference irradiation type/irradiation type under investigation), which is inversely related to a given dose (Equation (3) in Material and Methods) [13,23]. Generally, the higher the deposited energy, the higher the density of ionizing events, and the higher the resulting RBE per unit of dose, as defined by more severe DNA damage [17,24].

However, it is increasingly appreciated that not only the physical characteristics of the beam but also the microscopic pattern of energy deposition differs between photons and protons, particularly at the distal edge of the Bragg-peak, with a potential impact on the resulting biological effects [14,20]. In fact, depending on the tissue, the measured endpoint, dose and LET of the beam, the RBE values reported for protons vary between ~ 1.1–1.7, with increasing RBE values for protons along the distal edge of the Bragg peak [18,21,25,26,27]. Moreover, first in vitro studies implicated that cancer-associated genetic defects in DNA repair—homologous recombination repair (HRR) or the Fanconi Anemia (FA) pathway—are associated with an increase in the RBE values for irradiation with proton beams compared to irradiation with gamma-ray photons or X-ray photons [28,29,30,31]. These observations point to potential differences in the biology of DNA damage induced by irradiation with proton beams compared to irradiation with gamma-ray photons or X-ray photons. Moreover, these findings suggest that genetic alterations affecting DNA damage response (DDR) and DNA repair pathways may not only contribute to heterogeneity in cancer cell radiosensitivity per se, but might also cause variations in proton RBE [22].

Independent of the radiation quality, the cytotoxic effects of ionizing radiation are based on its ability to cause damage to cellular macromolecules, particularly the DNA. Herein, DNA double strand breaks (DSB) are considered to be the most toxic lesions induced by ionizing radiation so that the capacity of cells to repair radiation-induced DNA DSB constitutes a major determinant of cellular radiosensitivity [32,33]. Radiation-induced DNA DSB are mainly repaired by non-homologous end joining (NHEJ) and HRR [34]. However, if both pathways are disrupted, alternative end joining (alt-EJ) can be activated [35,36]. NHEJ is a cell cycle independent and rapid, but error-prone DNA repair pathway that is mediated by the DNA-PK (DNA-dependent protein kinase) complex consisting of the KU70/80 heterodimer and a catalytic subunit (DNA-PKcs) [34]. DNA-PKcs is essential for the recruitment of repair proteins of the NHEJ complex, including XRCC4 (X-ray repair cross-complementing protein 4), Lig IV (DNA ligase IV), the nuclease Artemis as well as the stabilizing factors XLF (XRCC4-like factor) and PAXX (paralog of XRCC4 and XLF) [37,38,39,40]. In contrast, HRR is a precise, but slow and cell cycle-dependent DNA repair mechanism requiring homology of a sister chromatid [34,41,42]. HRR efficiency relies on numerous proteins and protein complexes, e.g., BRCA2 (breast cancer 2) and Rad54. BRCA2 is a tumor suppressor and one of the most essential proteins regulating HRR; it promotes binding of Rad51 to single-stranded DNA during DNA damage processing [43]. Rad54 induces DNA synthesis and promotes dissociation of Rad51 from the DNA strand, ensuring annealing of the synthesized DNA to the ends of the damaged DNA strand [44]. In this context, 53BP1 (p53 binding protein 1) functions as a sensor of DNA damage and has been proposed to mediate DNA repair pathway choice by promoting NHEJ [44,45]. 

Though an increasing number of reports point to different patterns of DNA damage induced by photon and proton irradiation with potential relevance for DNA repair [14,28,29,30,46] there is a gap of knowledge about potential microscopic differences in the DNA damage pattern induced by protons from the entrance plateau (EP) versus protons from SOBP. Moreover, only limited data are available on variations in RBE caused by genetic deficiencies in components of the two major DNA DSB repair pathways, NHEJ and HRR, which are also observed in human cancer. Such investigations are particularly important in view of the increasing interest in combining DNA repair pathway inhibitors with radiotherapy for inducing synthetic lethality in tumors with intrinsic or acquired DNA repair-deficiencies [47,48,49,50].

In the present study, we used STED (stimulated emission depletion) microscopy to compare the microscopic pattern of DNA damage induced by irradiation with equal physical doses of SOBP protons, EP protons and X-ray photons. Moreover, we used fibroblasts and cancer cells without and with deficiencies in specific proteins of NHEJ, HRR or both, to explore potential differences in RBE upon irradiation with a similar physical dose of protons (SOBP, EP) and X-ray photons for the endpoints clonogenic cell survival and DNA DSB repair kinetics. We observed the induction of more clustered γH2A.X foci in cells exposed to irradiation with SOBP protons. Moreover, we noticed a significant increase in cell death in fibroblasts and cancer cells with deficiency in a single core protein of NHEJ upon irradiation with both protons and photons. Instead, MEFs with deficiency in a single core protein of HRR displayed enhanced radiosensitivity to proton irradiation but not to X-ray photons when compared to the respective HRR-proficient MEF cells. 

Further comprehensive work in additional repair-deficient cell lines and patient-derived cancer cell lines are required to define signaling molecules specifically enhancing RBE values upon irradiation with proton beams. Such investigations are needed to provide a scientific basis for the identification of patients that might particularly benefit from proton beam radiotherapy according to the molecular characteristics of their tumors for the definition of combinatorial treatments suited to harness the full potential of radiotherapy with SOBP protons whilst avoiding increased toxicity.

## 2. Materials and Methods 

### 2.1. Chemicals and Antibodies

Alexa Fluor 647-coupled antibody against γH2A.X protein was obtained from Becton Dickinson (Heidelberg, Germany). Anti-53BP1 rabbit polyclonal antibody was purchased from Santa Cruz (Heidelberg, Germany). Secondary antibody Alexa Fluor 555 (anti-rabbit) and Hoechst33342 were purchased from Invitrogen (Eugene, OR, USA). DAKO Fluorescent mounting medium from Dako North America Inc. (Carpinteria, CA, USA) was used. All media, fetal bovine serum (FBS) and penicillin-streptomycin (pen/strep) were acquired from Thermo Fisher Scientific (Waltham, MA, USA). All other chemicals were purchased from Sigma-Aldrich (Deisenhofen, Germany) unless otherwise specified.

### 2.2. Cell lines and Cell Culture

M059K (DNA-PKcs-proficient) and M059J (DNA-PKcs-deficient) glioblastoma cell lines [51], wild type MEFs (murine embryonic fibroblasts) and MEFs with genetic deficiency in DNA repair proteins (Rad54^−/−^, Lig IV^−/−^, Rad54^−/−^/Lig IV^−/−^), Capan-1 (human pancreatic adenocarcinoma BRCA2^−/+^ 6174delT) and BxPC3 (human pancreatic adenocarcinoma) [52], 2BN hTERT human fibroblasts XLF^−/−^ and control fibroblasts [53], RPE-1 human retinal PAXX-deficient (PAXX^−/−^) and control cells [40] were kindly provided by Prof. Dr. George Iliakis (Institute of Medical Radiation Biology, University of Duisburg-Essen, Germany). 2BN hTERT control and XLF-deficient (XLF^−/−^) cells were cultured in MEM supplemented with 10% FBS (fetal bovine serum), non-essential amino acids and 1% pen/strep. RPE-1 control and PAXX^−/−^ cells were cultured in DMEM/F12 supplemented with 10% FBS and 1% pen/strep. All other cell lines were cultured in DMEM supplemented with 10% FBS and 1% pen/strep. All cell lines were routinely checked for mycoplasma contamination.

### 2.3. Irradiation

**Figure 1:**
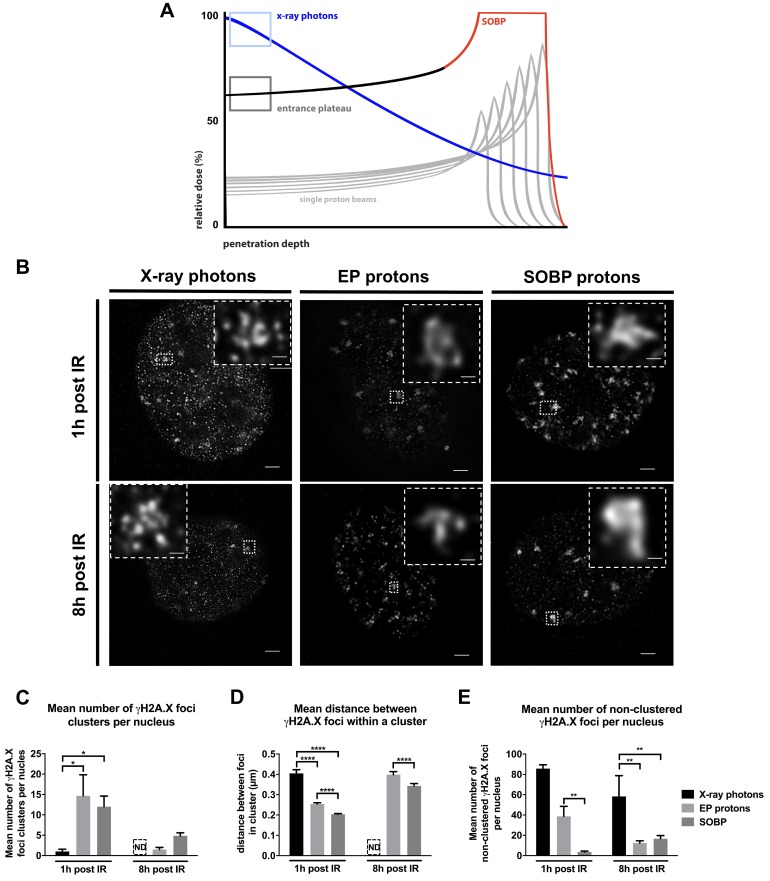
Time-dependent induction and removal of γH2A.X foci in MEFs irradiated with a physical dose of 3 Gy of X-ray photons, EP and SOBP protons visualized by γH2A.X staining. (**A**) Model of irradiation setup. Depth-dose curves of an X-ray photon beam and a proton beam highlighting the depth area where the cells were irradiated with X-ray photons, Bragg-peak protons and plateau protons. (**B**) STED microscopy pictures of a single nucleus of MEFs after 1 h and 8 h of 3 Gy irradiation with X-ray photons, EP protons or SOBP protons (objective 100×); scale bar: 0.1 µm. Clustered γH2A.X foci were additionally zoomed in 40× (small squares in the edge). Scale bar: 0.1 µm. (**C**–**E**) Quantification of γH2A.X foci in MEFs irradiated with 3 Gy of X-ray photons, EP and SOBP protons. (**C**) Average number of clusters per nucleus. (**D**) Average distance between γH2A.X foci in clusters. (**E**) Average number of foci per nucleus beyond clusters. Data represent mean ± SEM; One-way ANOVA with Tukey’s multiple comparisons post-test and Kruskal–Wallis test; * *p* < 0.05; ** *p* < 0.01; **** *p* < 0.0001; ND – not detectable.

Cells were exposed to an equal physical dose of 3 Gy for γH2A.X assay or 1, 2, 4, 6 and 8 Gy for long-term clonogenic survival assays, independent of different beams and irradiators used. We present the model of irradiation in Figure 1A and show the distribution of relative dose in percent. 

#### 2.3.1. X-ray Photon Irradiation

X-ray photon irradiation by X-RAD 320 X-Ray Biological Irradiator with a MIR-324 X-ray tube (Precision X-Ray Inc., North Branford, CT, USA) 3.75 Gy/min at a distance of 50cm from the X-ray tube window was controlled by a parallel dosimetry with the PTW 7862 parallel plate transmission chamber and PTW UNIDOS dosimeter (Precision X-Ray Inc., North Branford, CT, USA).

#### 2.3.2. Proton Irradiation

Proton irradiation was performed on a Proteus Plus with a 230 MeV cyclotron (IBA International, Louvain-La-Neuve, Belgium). The plates with cell monolayers covered with 2 ml of culture medium for 12-well and 6-well plates were placed on a treatment table and irradiated in pencil beam mode in a defined source axis distance in the isocenter. Cells were exposed to either mid SOBP or EP proton irradiation. A narrow SOBP was necessary to account for uncertainties in range and scattering as well as exact cell positions while maintaining the SOBP region. The maximum energy of 110 MeV (range approx. 9 cm in water) and the lowest energy of 100 MeV (range approx. 7.6 cm in water) of the SOBP (in total six layers) must therefore be transmitted through a range shifter (thickness 7.4 cm). The range shifter offers the possibility to reach the desired measuring depth. A 2 mm solid plate phantom was used as build up to position the cells in the EP region of the depth dose curve. SOBP was composed of 6 single Bragg peaks with following energies in MeV: 1: 109.9; 2: 107.6; 3: 105.1; 4: 103.1; 5: 100.9; 6: 100. To achieve the same dose for the EP proton region as for SOBP, the range shifter was not applied, and the time of irradiation was increased.

Irradiation fields were created and optimized by the clinical planning system and calibrated by measuring the dose with a 2D array detector MatriXX PT (IBA International, Louvain-La-Neuve, Belgium) at the same depth as the cells were placed during the irradiation.

### 2.4. Colony Formation Assay

Clonogenic cell survival was tested in response to ionizing radiation with doses between 1 and 8 Gy as previously described [54]. Exponentially grown cells were seeded in 6-well plates and were irradiated 24 h later. For determination of colony formation, cells were fixed after 7–10 days in 3.7% formaldehyde and 70% ethanol, and stained with 0.05% Coomassie blue. Colonies of at least 50 cells were counted. Survival data were calculated using the linear-quadratic model and the following equation:

S_(D)_ = exp [− (αD + βD^2^)] (1)

where S_(D)_ – survival fraction probability at a given radiation dose (D), α – linear and β – quadratic parameter of cells’ radiosensitivity [55].

The linear (α) and quadratic (β) parameters were calculated for each survival curve form, stratified and fitted to the liner-quadratic model colony formation survival data. The dose D_(S)_ to achieve a given survival level (S) was calculated using transformed Equation (1):

D_(S)_ = − (α/2β) ± [0.25(α/β)^2^ − (ln(S)/β)]^0.5^ (2)

The RBE values were calculated as previously described using Equation (3):

RBE_(S)_ = D_(S) X-rays_/D_(S) particle_ (3)

where RBE_(S)_ – RBE at a given cell survival level (10%), D_(S) X-rays_ – dose of X-ray photons and D_(S) particle_ – dose of EP/SOBP protons required to achieved given cell survival (S) [23,56]. 

### 2.5. Immunofluorescence Staining

Cells were fixed and permeabilized with 3% paraformaldehyde (PFA) and 0.2% Triton X-100 in PBS for 15 min at indicated time points after irradiation. After washing with PBS, cells were blocked overnight with 2% goat serum in PBS. Antibodies were diluted in blocking buffer. Incubation with antibody against 53BP1 was performed for 1 h in a 1:100 dilution. Alexa Fluor 647-conjugated anti-γH2A.X antibody was incubated for 1 h at a 1:100 dilution. Staining with secondary antibody - Alexa Fluor 555 (anti-rabbit) was performed in the dark for 1 h at a dilution 1:400. Samples were washed after each incubation step three times with PBS followed by staining for 15 min in the dark with 0.2% (*w/v*) Hoechst33342 diluted in PBS. Samples were again washed with PBS, mounted with the DAKO mounting medium and stored at 4 °C in the dark. Single layer fluorescence images were taken with a Zeiss AxioCam MRm (1388 × 1040 pixels) at a Zeiss Axio Observer Z1 fluorescence microscope with Plan-Apochromat 63×/1.40 Oil M27 lens, 49 DAPI, 38 HE, 43HE and 78 HE ms filter and a transmission grid VH “ApoTome” (Carl Zeiss, Goettingen, Germany). Images were taken with three quarters of the maximum intensity without overexposure. The pictures were saved as 16-bit multi-channel Carl Zeiss Image files (CZI) with no further editing. Foci were analyzed with the Focinator software [57,58]. Software, instructions and supporting information are provided at https://www.focinator.com.

For super resolution STED microscopy, 100,000 cells were seeded in full growth medium on #1.5 thickness high precision (18 mm × 18 mm) coverslips (Scientific Thermo Fisher; Waltham, MA, USA), placed in standard 6-well plates, and irradiated 24 h after seeding with 3 Gy of either photon or SOBP proton. Cells were fixed and permeabilized (3% paraformaldehyde (PFA) and 0.2% Triton X-100 in PBS; 15 min; RT). After washing with PBS, cells were blocked overnight with 2% goat serum in PBS. γH2A.X antibody was diluted in blocking buffer. Incubation with antibody against γH2A.X was performed for 1 h in a 1:100 dilution. Alexa-Fluor 488 (anti-rabbit) diluted 1:100 in 2% goat serum was incubated for 1 h in dark. Mounting was performed using the TDE mounting medium [59].

### 2.6. Superresolution STED Microscopy

Gated stimulated emission depletion (STED) microscopy was performed using a Leica TCS SP8 epifluorecence-confocal-microscope equipped with a picosecond pulsed white light laser for excitation at 488 nm and a 592 nm STED depletion laser (Leica, Wetzlar, Germany). STED confocal images were acquired with a pixel resolution of 21 nm using the Leica HCX PL APO 100×/1.4 Oil STED objective. These images were later deconvolved using the STED deconvolution option of Huygens Professional (v. 16.05, SVI, Hilversum, Netherlands), which resulted in a final transverse resolution of 76.22 nm +/− 3.47 nm (*n* = 6). The coordinates and number of foci was measured by intensity of foci using ImageJ. From the foci coordinates a nearest neighbor analysis was performed with a script written in R using the spatial statistics package ‘spatstat’ [60].

### 2.7. Alkaline Single Cell Gel Electrophoresis (Comet) Assay

Exponentially growing cells cultured in 6-well plates were irradiated with 8 Gy photon or proton irradiation. Alkaline buffers were prepared according to [61]. Cells were collected at defined time points after irradiation (30 min, 4 h, 8 h, 24 h) by trypsinization. The cell suspension (100 μL) was mixed with 200 μL agarose (40 °C, 1% in ddH_2_O, low melting, Sigma-Aldrich, Deisenhofen, Germany) and pipetted onto an agarose-precoated slide. The agarose clot was covered with a cover slip, which was removed after solidification of the agarose and subsequently placed in alkaline lysis buffer (pH > 13.0) for 1 h. The slides were then placed in alkaline electrophoresis buffer (pH > 12.3) for 10 min to allow buffer exchange prior to electrophoresis for 1 h (0.75 V/cm electrode distance). Subsequently, the slides were transferred in ddH2O for 10 min and allowed to air-dry overnight at room temperature. For fluorescence microscopy, propidium iodide solution (50 μg/mL in ddH2O) was added onto the dried slide and sealed with a cover slip. The comets were imaged by the Axio Scanner Z.1 with 10× objective magnification and using the ZEN 2 Blue edition software (Carl Zeiss AG, Germany) and the tail length determined with ImageJ (1.51j8 available at https://imagej.nih.gov/ij/; U.S. National Institutes of Health, Bethesda, MD, USA) and the plugin OpenComet (OpenComet v1.3.1) [62].

### 2.8. Statistical Analysis and Reproducibility 

Data represent mean values of at least three independent experiments. Data were first tested for normal distribution using the D’Agostino–Pearson omnibus normality test. Further, data analysis was performed by standard one-way ANOVA test with Tukey two-pair comparison post-test for normally distributed values or non-parametric Kruskal–Wallis test for values lacking normal distribution. Two-way ANOVA test with Tukey’s or Sidak’s multiple comparison post-tests were applied for statistical calculation of the influence of two different parameters. Calculations were done using Prism 6^TM^ software (GraphPad Inc., La Jolla, CA, USA). *p*-values ≤ 0.05 were considered as statistically significant and indicated as a star symbol where * *p* ≤ 0.05, ** *p* < 0.01, *** *p* < 0.001 and **** *p* < 0.0001.

## 3. Results

### 3.1. Proton Irradiation Induces Larger DNA repair γH2A.X Foci when Compared to Irradiation with X-ray Photons

To explore suggested differences in the biology of DNA damage induced by proton and photon irradiation, we exposed in vitro cultured cells to irradiation with a physical dose of 3 Gy generated by X-ray photons, mid SOBP protons (SOBP composed of 6 single Bragg peaks) and EP protons (Figure 1A). First, we irradiated MEFs and analyzed the distribution of γH2A.X foci in the nucleus 1 h and 8 h after irradiation using a STED super resolution microscopy (Figure 1B). While X-ray photon irradiation mainly resulted in the formation of small γH2A.X foci equally distributed within the nucleus, SOBP protons created a more heterogeneous foci distribution with a high number of larger γH2A.X foci (Figure 1B).

STED microscopy analysis of γH2A.X foci upon SOBP proton irradiation revealed that the pattern of DNA damage foci consisted of several small sub clusters of γH2A.X foci in close proximity to each other and with larger distance to neighboring γH2A.X foci accumulations yielding a pattern of larger cluster formations within the nucleus; we therefore termed the γH2A.X foci with the larger appearance “foci clusters” throughout the manuscript (Figure 1B). Instead, spatial distribution of foci upon irradiation of the MEFs with EP protons, was characterized by a mixture of both types of DNA damage foci described above, namely homogenously distributed small foci, as well as heterogeneously distributed foci clusters (Figure 1B). ImageJ-based quantitative analysis of STED pictures confirmed a significantly higher number of γH2A.X foci clusters after irradiation with SOBP or EP protons at 1 h post-irradiation than after X-ray photon irradiation, where we barely observed γH2A.X foci clusters (Figure 1C). Over time, the number of clustered γH2A.X foci declined significantly, or clusters were no longer detectable. 

For STED analysis, we picked spots of accumulated γH2A.X foci and used those for a more detailed analysis. To better define the features of clusters, we determined the distance between single γH2A.X foci within each cluster. As shown in Figure 1D, the distance between foci was significantly lower in SOBP- and EP-induced clusters. However, this distance increased over time, pointing to a time-dependent removal of single γH2A.X foci indicative of ongoing DNA repair (Figure 1D). 

Besides the number of γH2A.X foci clusters, we determined the number of non-clustered γH2A.X foci (Figure 1E). Expectedly, X-ray photon irradiation caused the highest level of equally distributed non-clustered DNA damage foci (Figure 1E). Interestingly, irradiation with EP protons evoked a significantly higher number of non-clustered γH2A.X foci compared to SOBP proton irradiation (Figure 1E). However, the equally distributed DNA damage was mostly resolved within 8 h after EP proton irradiation but was still highly present in nuclei irradiated by X-ray photons (Figure 1E). Taken together, these findings on the spatial distribution of γH2A.X foci suggest that irradiation with SOBP protons evokes more clustered DNA damage when compared to X-ray photons, whereas EP protons induce both, SOBP proton-like and X-ray photon-like DNA damage distribution.

### 3.2. Deficiency in Lig IV Strongly Sensitizes MEFs to Irradiation with Proton Beams and X-ray Photons 

**Figure 2:**
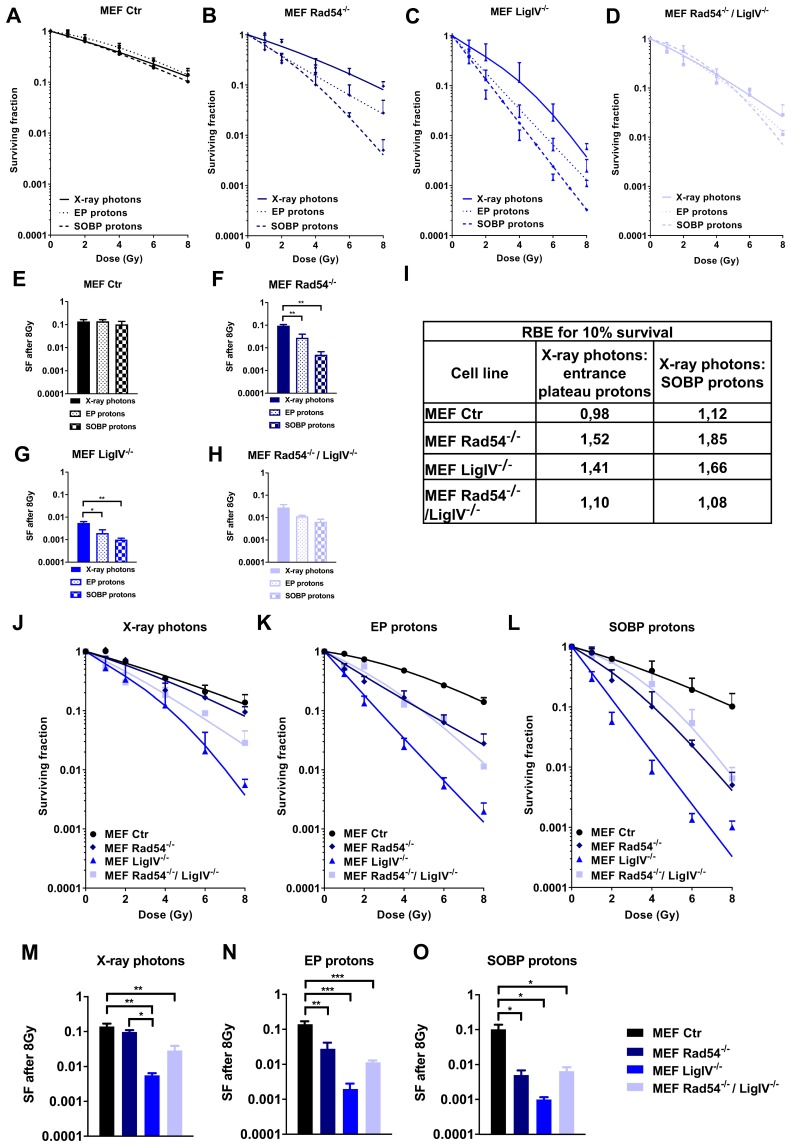
Effects of X-ray photons, EP and SOBP protons on clonogenic cell survival. (**A**–**H**) Results of colony formation assay in wild type MEFs (Ctr) and MEFs harboring genetic deficiency (^−/−^) in Rad54 (Rad54^−/−^), Lig IV (Lig IV^−/−^) or Rad54 and Lig IV (Rad54^−/−^/Lig IV^−/−^), upon irradiation with X-ray photons, EP protons or SOBP protons (1–8 Gy) as indicated. (**I**) RBE values of clonogenic survival calculated for 10% survival of the indicated MEF cell lines. (**J**–**L**) Clonogenic survival curves of MEFs upon irradiation with 1–8 Gy of X-ray photons (**J**), EP protons (**K**) or SOBP protons (**L**). (**M**–**O**) Comparison of cell survival between MEFs harboring distinct DNA repair protein-deficiencies as described above upon irradiation with 8 Gy of X-ray photons (**M**), EP protons (**N**) or SOBP protons (**O**). Data represent mean values ± SD (**A**–**D**,**J**–**L**) or ± SEM (**E**–**H**,**M**–**O**) of three independent experiments. One-way ANOVA with Tukey’s multiple comparisons post-test; * *p* < 0.05; ** *p* < 0.01; *** *p* < 0.001.

The distinct spatial distribution of γH2A.X foci upon irradiation with protons and X-ray photons observed in our STED analysis pointed to differences in biology of the DNA damage induced by different irradiation sources. This prompted us to explore whether the observed variations in the biology of the damage might result in distinct radiosensitivity of cells depending on the applied radiation quality and cellular proficiency in DNA repair. To this end, we first compared clonogenic survival of wild type MEFs after exposure to irradiation with X-ray photons, EP protons or SOBP protons, respectively. However, we did not observe significant differences in clonogenic survival of wild type MEFs exposed to the different radiation qualities (Figure 2A,E), revealing a comparable sensitivity of MEFs to irradiation with X-ray photons and protons. Next, we explored potential differences in the sensitivity of cells with defects in DNA repair pathways to proton vs. X-ray photon irradiation, since others suggested differences in DNA damage response in cells with deficiencies in HRR or FA [28,29,30]. Therefore, we compared clonogenic survival of MEFs with defects in specific DNA repair proteins, including Lig IV^−/−^ (NHEJ), Rad54^−/−^ (HRR) or both, Lig IV^−/−^ and Rad54^−/−^, upon irradiation with a similar physical dose of X-ray photons, EP protons or SOBP protons (Figure 2B–D,F–H). In contrast to wild type MEFs, MEF cell lines with a deficiency in Lig IV (MEF Lig IV^−/−^) or Rad54 (MEF Rad54^−/−^) turned out to be significantly more sensitive to irradiation with protons compared to X-ray photons, as depicted exemplarily for 8 Gy (Figure 2F–G). Of note, values for double-deficient MEF Rad54^−/−^/Lig IV^−/−^ did not reach statistical significance (Figure 2H). Interestingly, none of the tested cell lines showed a significant difference in clonogenic survival after irradiation with EP or SOBP protons (Figure 2E–H).

Further, comparing the responses of wild type MEFs and the DNA repair-deficient MEFs to irradiation with X-ray photons, EP protons or SOBP protons, we found that deficiency in Lig IV exerted the most pronounced effect on clonogenic survival, irrespective of the radiation quality used (Figure 2J–L). 

Moreover, while single loss of Rad54 failed to significantly affect survival of MEFs upon X-ray photon irradiation, loss of Lig IV alone or both, Lig IV and Rad54, significantly increased the sensitivity of MEFs to irradiation with X-ray photons compared to wild type MEFs (Figure 2M). Loss of Lig IV also significantly increased the radiosensitivity to X-ray photons compared to single loss of Rad54 (Figure 2M). However, loss of Rad54, Lig IV, or both rendered MEFs significantly more sensitive to irradiation with 8 Gy of either EP protons or SOBP protons (Figure 2N,O). Here, Rad54^−/−^/LigIV^−/−^ double knockout MEFs displayed similar survival to Rad54^−/−^ single knockout MEFs revealing that additional loss of Lig IV did not render Rad54-deficient MEFs more sensitive to proton irradiation (EP or SOBP) compared to Rad54-deficiency alone (Figure 2N,O).

To correlate variations in the efficiency of EP and SOBP proton irradiation to the different DNA repair-deficiencies, we subsequently calculated variations in RBE for 10% of clonogenic survival. The highest RBE values (1.84 and 1.66) were achieved after SOBP proton irradiation in Rad54^−/−^ and Lig IV^−/−^ MEFs, respectively (Figure 2I). In general, RBE values for SOBP protons were higher than those determined after EP proton irradiation. 

To explore if deficiency in other proteins with relevance to NHEJ might reproduce the strong effects of Lig IV-deficiency on the survival of irradiated fibroblasts, we included two immortalized fibroblast cell lines deficient in XLF and PAXX in our investigations. However, neither deletion of XLF (Figure S1A,B) nor PAXX (Figure S1F,G) significantly affected long-term survival of MEFs after irradiation with X-ray photons and SOBP protons. However, XLF-deficiency slightly radiosensitized MEFs to EP proton irradiation at 8 Gy of dose leading to a higher RBE for EP proton irradiation (Figure S1B,C). Unexpectedly, SOBP proton irradiation was less efficient than X-ray photon irradiation in RPE-1 control cells resulting in a lower RBE of 0.7 (Figure S1H). 

### 3.3. Lig IV is Essential for the Repair of DNA Damage Induced in MEFs by Irradiation with X-ray Photons and SOBP Protons

To gain insight into the relative importance of the above-mentioned NHEJ and HRR proteins for the repair of DNA damage induced by irradiation with X-ray photons vs. SOBP protons, we next determined potential differences in DNA damage induction and time-dependent removal of γH2A.X and 53BP1 foci in wild type MEFs as well as in MEFs harboring deficiencies in Lig IV, Rad54 or both, Lig IV and Rad54. Since we have not observed substantial differences in clonogenic survival of MEFs between irradiation with SOBP and EP protons (Figure 2), we focused on SOBP protons in our further experiments. 

**Figure 3:**
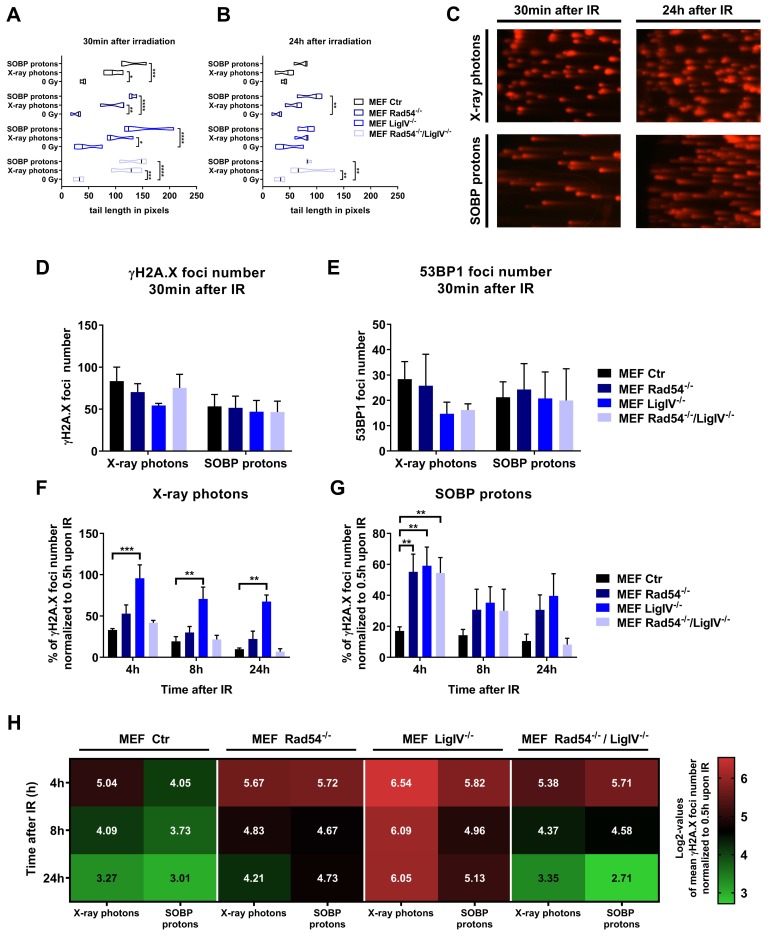
DNA damage repair induced by X-ray photons or SOBP protons in MEFs. (**A**,**B**) Quantification of alkaline comet assay in wild type (Ctr), Lig IV-deficient (Lig IV^−/−^), Rad54-deficient (Rad54^−/−^) or Lig IV/Rad54 double-deficient MEFs (Rad54^−/−^/Lig IV^−/−^) at 30 min (**A**) and 24 h (**B**) after irradiation with 3 Gy of X-ray photons or SOBP protons. (**C**) Exemplary pictures of alkaline Comet assay of wild type MEFs (MEF ctr) 30 min or 24 h after X-ray photon or SOBP proton irradiation. (**D**) γH2A.X and (**E**) 53BP1 foci number 30 min after irradiation with 3 Gy of X-ray photons and SOBP protons. (**F**,**G**) Quantification of DNA repair kinetics of MEFs harboring deficiencies in Rad54, Lig IV or Rad54 and Lig IV determined by quantification of γH2A.X foci. Data represent values normalized to 30 min after irradiation with 3 Gy. (**H**) Comparison of radiation source effects in tested cell lines as log2 values of γH2A.X foci quantification. Data represent mean SEM from three independent experiments. Two-way ANOVA with Tukey’s multiple comparisons post-test; * *p* < 0.05; ** *p* < 0.01; *** *p* < 0.001; **** *p* < 0.0001.

Quantification of DNA damage by using the alkaline comet assay demonstrated that exposure to X-ray photons and SOBP protons significantly increased DNA damage in wild type MEFs as well as in MEFs deficient in NHEJ or HRR proteins at 30 min after irradiation compared to non-irradiated control cells (Figure 3A,C left panel). Interestingly, the initial overall amount of DNA damage induced by SOBP protons was slightly increased when compared to X ray photons, independent of the cell line (Figure 3A,C left panel). However, deficiency in Lig IV, Rad54, or both, did not significantly alter the overall amount of initial DNA damage induced by SOBP protons vs. X-ray photons at 30 min after irradiation (Figure 3A,C left panel). To corroborate these findings, we additionally used the γH2A.X and 53BP1 foci staining as a marker for DNA damage induction. However, we did not detect significant differences in the number of initial foci between tested cell lines when using X-ray photons vs. SOBP protons (Figure 3D,E). 

However, despite similarities in the amount of initial DNA damage, cells with genetic deficiencies in DNA repair proteins may differ in the capacity for and kinetics of repair of radiation-induced DNA damage. Therefore, we additionally quantified the amount of residual DNA damage 24 h after irradiation. As depicted in Figure 3B,C (right panel), the highest amount of residual DNA damage 24 h after irradiation with SOBP protons was observed in Rad54^−/−^ MEFs, followed by Lig IV^−/−^ MEFs and Rad54^−/−^/Lig IV^−/−^ MEFs (Figure 3B,C right panel), though the double-deficient MEFs were characterized by an accumulation of residual DNA damage after irradiation with both, X-ray photons and SOBP protons (Figure 3B,C right panel). Next, we compared the capacity of wild type MEFs as well as of MEFs with genetic deficiencies in DNA repair proteins for the time-dependent removal of γH2A.X foci after irradiation with X-ray photons (Figure 3F) and SOBP protons (Figure 3G). Of note, when irradiation was performed with X-ray photons, only loss of Lig IV caused a significant delay in the removal of γH2A.X foci at 4 and 8 h after irradiation and an accumulation of residual γH2A.X foci at 24 h after irradiation. In contrast, MEFs deficient in Rad54 or both, Rad54 and Lig IV, displayed almost similar DNA repair kinetics as wildtype MEFs (Figure 3F). However, upon irradiation with SOBP protons, a significant delay in the removal of γH2A.X foci was observed in all cell lines with deficiency in NHEJ or HRR proteins when compared to wild type MEFs, at least at 4 h after irradiation (Figure 3G). 

Taken together, while deficiency in Lig IV delayed DNA repair kinetics after irradiation with both, X-ray photons and SOBP protons compared to wild type MEFs, the removal of γH2A.X foci was only delayed by deficiency in Rad54 or both, Rad54 and Lig IV^−/−^, compared to wild type MEFs when irradiation was performed with SOBP protons (Figure 3F,G). Moreover, a direct comparison between the response of the different MEFs to X-ray photons and SOBP protons further revealed that the most pronounced difference in the removal of γH2A.X foci between X-ray photon and proton irradiation was observed in Rad54^−/−^ MEFs, pointing to a more pronounced dependency of MEFs on integrity of HRR proteins when irradiated with SOBP protons than with X-ray photons (Figure 3H). An opposite trend was observed in Lig IV^−/−^ MEFs where the removal of γH2A.X foci was slightly more efficient upon X-ray photon irradiation (Figure 3H). 

In line with the findings obtained with Lig IV-deficient MEFs, XLF-deficiency significantly delayed DNA repair kinetics after irradiation with both X-ray photons (Figure S1D) and SOBP protons (Figure S1E) in the 2BN fibroblast cell line. As expected, PAXX-deficiency had no effect on the removal of radiation-induced γH2A.X foci (Figure S1I,J). Moreover, a direct comparison of time-dependent γH2A.X foci removal in XLF^−/−^ cells and the respective control cells revealed a difference after both X-ray photon and SOBP proton irradiation (Figure S1K). Again, PAXX-deficiency had no effect on γH2A.X foci removal after X-ray photon irradiation; however, we noticed a trend towards a higher amount of γH2A.X foci after SOBP proton irradiation (Figure S1L).

### 3.4. Impairment of NHEJ or HRR shows a Different Impact on Sensitivity of Cancer Cells to Irradiation with X-ray Photons or Protons

**Figure 4:**
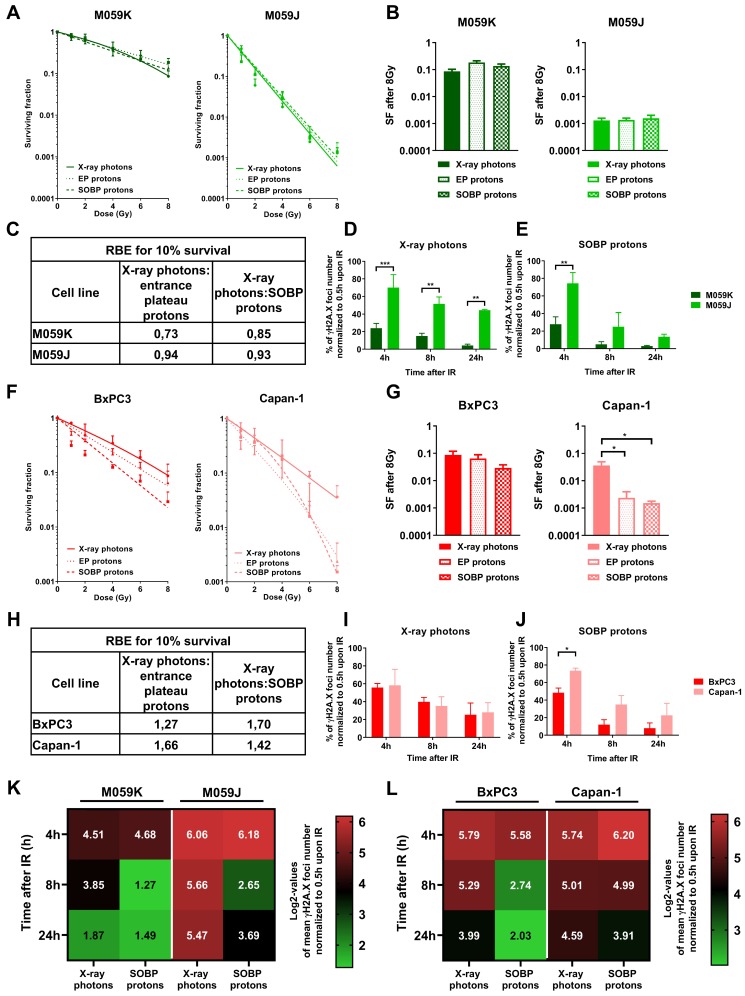
Effects of X-ray photons, EP and SOBP protons on clonogenic cell survival and kinetics in formation and removal of γH2A.X foci in cancer cells. (**A**,**B**) Clonogenic survival assay of DNA-PKcs-deficient M059J and DNA-PKcs-proficient M059K glioblastoma cells irradiated with X-ray photons, EP and SOBP protons (1–8 Gy). (**C**) RBE values calculated for 10% of clonogenic cell survival of the indicated cell lines. (**D**,**E**) γH2A.X foci removal over time (4–24 h) normalized to initial foci number at 30 min in M059J and M059K cells upon irradiation with 3 Gy of X-ray photons (**D**) and SOBP protons (**E**). (**F**,**G**) Clonogenic survival assay of pancreatic cancer cells with BRCA2-deficiency (Capan-1) and BRCA2-proficiency (BxPC3). (**H**) RBE values calculated for 10% of clonogenic cell survival of the indicated cell lines. (**I**,**J**) γH2A.X foci removal over time (4–24 h) normalized to initial foci number at 30 min in Capan-1 and BxPC3 cells upon irradiation with 3 Gy of X-ray photons (**I**) and SOBP protons (**J**). (**K**,**L**) Comparison of radiation quality effects in tested cell lines as log2 values of γH2A.X foci quantification. Data represent mean values ± SD (**A**,**F**) or ± SEM (**B**,**D**,**E**,**G**,**I**,**J**) from three independent experiments. One-way ANOVA with Tukey’s multiple comparisons post-test (**B**,**G**) or two-way ANOVA with Tukey’s multiple comparisons post-test (**D**,**E**,**I**,**J**); * *p* < 0.05; ** *p* < 0.01; *** *p* < 0.001.

So far, our data indicated that deficiency in NHEJ and HRR proteins has a different impact on the repair of radiation-induced DNA damage and survival of normal MEFs. However, ionizing radiation is mostly used to treat cancer. We thus next investigated if we observe similar differences in cancer cells with deficiency in proteins relevant to NHEJ or HRR. To this end, we used glioblastoma cells proficient (M059K) and deficient (M059J) in DNA-PKcs, as well as pancreatic cancer cells without (BxPC3) and with a naturally occurring 6174delT mutation in one BRCA2-allele accompanied by loss of the wild-type allele (Capan-1). Similar to the investigations in the MEFs, we irradiated cancer cells with a similar physical dose of X-ray photons, SOBP protons, or EP protons (Figure 4A,B,F,G). Deficiency in DNA-PKcs caused a dramatic radiosensitization independent of the radiation quality used (Figure 4A). Of note, comparison of different irradiation sources in DNA-PKcs-deficient vs. DNA-PKcs-proficient cells at a dose of 8 Gy revealed no difference in clonogenic survival (Figure 4B). As a consequence of the very high sensitivity of the DNA-PKcs-deficient cells to irradiation with X-ray photons, the RBE values for clonogenic cell survival turned out to be rather similar for all 3 radiation qualities in both cell lines, since irradiation with X-ray photons served as reference radiation quality (Figure 4C). Interestingly both, BRCA2-proficient BxPC3 and Capan-1 pancreatic cancer cells with BRCA2-deficiency turned out to be more sensitive to SOBP proton irradiation when compared to X-ray photon irradiation (Figure 4F,G). Though this effect was much more pronounced in Capan-1 cells with BRCA2-deficiency (Figure 4G).

BRCA2-deficient Capan-1 cells were slightly more sensitive to X-ray photon irradiation, when compared to BRCA2-proficient BxPC3 cells (Figure 4G). Importantly, we observed pronounced and significant differences in the sensitivity to irradiation with X-ray photons versus SOBP protons only in the BRCA2-deficient Capan-1 cells, whereas the BRCA2-proficient BxPC3 cells did not reach significant differences (Figure 4F,G). Furthermore, RBE values calculated for clonogenic cell survival of BxPC3 and Capan-1 cell lines after EP and SOBP proton irradiation were much higher than those determined for M059J and M059K cells. However, future investigations in a broader panel of pancreatic cancer cells lines are required to reveal a possible benefit of using proton irradiation for pancreatic cancer cells (Figure 4H). 

Next, we explored the effects of deficiency in DNA-PKcs and BRCA2 on the kinetics of DNA repair by measuring the time-dependent removal of γH2A.X foci in the respective cell lines (Figure 4E–H). Consistent with the results gained from the long-term survival experiments, the time-dependent removal of γH2A.X foci was significantly delayed in DNA-PKcs-deficient M059J cells when compared to M059K DNA-PKcs-proficient cells (Figure 4D,E). Interestingly, X-ray photons evoked a stronger delay in DNA repair kinetics on M059J cells than SOBP proton irradiation resulting in a higher amount of residual DNA damage at 24 h after irradiation (Figure 4D,E).

Removal of γH2A.X foci was rather similar in BxPC3 and Capan-1 cells upon irradiation with X-ray photons. In contrast, we observed a pronounced delay in time-dependent removal of γH2A.X foci in the BRCA2-deficient Capan-1 cells upon irradiation with SOBP protons compared to X-ray photon irradiation (Figure 4I,J). Instead, removal of γH2A.X seemed to be slightly more efficient in the BxPC3 cells upon SOBP proton irradiation, reaching significant values at the 4-h time point (Figure 4I,J). Yet, mechanistic investigations are required to explore whether genetic BRCA2-deficiency is causative for the observed differences in DNA repair kinetics and survival of Capan-1 cells between SOBP proton and photon irradiation.

To better visualize the effects of both irradiation modalities, X-ray photons and SOBP protons, on DNA repair kinetics of M059J and M059K as well as Capan-1 and BxPC3, we performed a direct comparison of normalized γH2A.X foci numbers at 4 h, 8 h and 24 h dependent on irradiation source (Figure 4K,L). First, a more pronounced delay in the time-dependent removal of γH2A.X foci compared to M059K cells, including the residual DNA damage after 24 h, was observed in M059J cells when using X-ray photon irradiation, whereas the effect was weaker upon SOBP proton irradiation (Figure 4K). Second, we only observed minor differences in DNA repair kinetics between the two radiation qualities in control cell line M059K (Figure 4K). On the other hand, removal of γH2A.X foci occurred with similar efficiency in BRCA2-proficient BxPC3 and BRCA2-deficient Capan-1 pancreatic cancer cells, resulting in similar DNA repair kinetics upon irradiation with X-ray photons (Figure 4L). Instead, removal of γH2A.X foci was slower in Capan-1 cells than in BxPC3 cells upon irradiation with SOBP protons (Figure 4L). This resulted in comparable levels of residual DNA damage foci in Capan-1 cells upon irradiation with X-ray photons and SOBP protons (Figure 4L). 

## 4. Discussion

While the physical characteristics of photon irradiation have been investigated in much detail during recent decades, potential specificities in biological effects of proton irradiation are less well understood. Dose-deposition profiles of therapeutic proton beams are characterized by a low-dose plateau at small depths EP and the SOBP. In the SOBP, the major part of energy is deposited shortly before the sharp distal energy fall-off. First, by using STED we reveal here that irradiation with X-ray photons resulted in homogenously distributed small DNA damage foci, whereas SOBP proton irradiation induced clusters of several smaller γH2A.X foci in closer proximity that we termed “foci clusters”. Interestingly, EP proton irradiation caused both clustered and homogenously distributed small γH2A.X foci within the nucleus of MEFs. Second, we demonstrated that loss of important components of HRR has a more severe impact on DNA repair kinetics and survival of cancer cells and MEFs exposed to a similar physical radiation dose of mid SOBP or EP protons than upon irradiation with X-ray photons. We speculate that the suggested increasing importance of repair by HRR in cancer cells irradiated with SOBP protons compared to X-ray photon irradiation might be due to the induction of more clustered DNA lesions composed of multiple DNA damage sites in close proximity, as demonstrated in MEFs by visualizing γH2A.X foci using STED microscopy. Yet further mechanistic investigations and investigations in patient-derived cells are needed to verify that cancer cells exposed to proton irradiation rely more on the integrity of the HRR pathway. 

In more detail, we first hypothesized that irradiation with protons (SOBP, EP) and X-ray photons may induce distinct microscopic patterns of DNA damage because the physical characteristics of proton beams include a higher LET and a deposition of more dose per path length than gamma-ray or X-ray photons [63]. Indeed, we observed that irradiation with SOBP protons induced γH2A.X DNA foci-clusters, whereas DNA damage foci induced by X-ray photons were smaller and more randomly distributed over the whole nucleus. These observations are consistent with the described action of particles, including protons, that induce a direct DNA damage within their defined track and therefore tend to induce accumulated DNA damage in closer proximity [13,14,64,65]. It has been previously suggested that the high energy of particles, e.g., protons, strongly correlates with clustered DNA damage in the form of coalesced DNA DSB due to denser ionizing events [6,13,14,64,66]. Moreover, a prediction analysis done by a new computational track structure model which simulates complexity of DNA damage after proton irradiation, endorses our assumption of a more complex DNA damage induced by proton irradiation [67]. Interestingly, we observed that EP proton irradiation caused both clustered and randomly distributed DNA damage. Therefore, we speculate that high energy protons of the EP region deposit their energy more randomly causing less ionizing events than the ‘precise’ SOBP protons and result therefore in lower probability to hit the cell nucleus and to induce only clustered DNA lesions [68]. Our findings support assumptions proposed by others that differences in dose deposition between photons and protons induce different DNA lesions. Thereby, more complex DNA damage induced by protons may result in less efficient repair and more effective eradication of clonogenic cells [14,28,29,46,69]. Furthermore, the time-dependent increase in distance between γH2A.X foci observed in our study suggests that the initial clusters are removed over time leaving smaller but longer-lasting foci [65,70].

Since X-ray photons, SOBP and EP protons induced different patterns of DNA damage, we further determined radiosensitivity of wildtype MEFs to all three radiation modalities and observed higher RBE values for 10% of clonogenic survival upon SOBP proton than upon EP proton irradiation. We therefore conclude that the effects of EP on survival are more similar to X-ray photons, at least as long as the MEFs have an intact DNA repair machinery (NHEJ, HRR). These observations corroborate findings obtained by others describing higher RBE values in a zebrafish model at mid SOBP compared to EP proton irradiation [71]. 

However, when analyzing MEFs harboring deficiencies in specific DNA repair proteins associated with NHEJ (Lig IV^−/−^), HRR (Rad54^−/−^) or both (Rad54^−/−^/Lig IV^−/−^), we observed divergent results. In fact, MEFs deficient in Lig IV turned out to be highly radiosensitive, independent of the radiation quality used. The high radiosensitivity of Lig IV-deficient MEFs was associated with high levels of residual DNA damage foci at 24 h after irradiation upon both photon and proton irradiation. These observations underline the importance of NHEJ for the DNA DSBs repair induced by both photon and proton irradiation. In contrast, Rad54^−/−^ MEFs responded only to proton irradiation with increased rates of clonogenic cell death, whereas they were similarly sensitive to photon irradiation as wild type MEFs. This is consistent with observations from others reporting that in cells with intact NHEJ a Rad54-deficiency is not associated with a detectable defect in DNA DSBs repair induced by high dose irradiation of X-ray photons [72]. The authors concluded that cells harboring NHEJ defects repair the majority of X-ray photon irradiation-induced DNA DSBs by using the slower alt-EJ, which is suppressed by NHEJ and mostly not impaired by mutations in HRR [72,73]. Nevertheless, for both cell lines, Lig IV^−/−^ and Rad54^−/−^, the RBE values determined for SOBP proton irradiation were higher than for EP, as observed in wildtype MEFs.

Interestingly, co-depletion of proteins involved in both NHEJ and HRR (Rad54^−/−^/LigIV^−/−^) rendered MEFs more sensitive to X-ray photon irradiation than a single depletion of Rad54^−/−^. Interestingly, at a higher dose of 8 Gy, no significant differences in radiation sensitivity were observed between LigIV^−/−^ single knockout and Rad54^−/−^/LigIV^−/−^ double knockout MEFs. Our results are consistent with previously published data [72], though higher doses of X-ray photon irradiation (10 Gy, 20 Gy) has been used in this study. Moreover, the authors concluded that Rad54-dependent HRR does not facilitate repair of radiation-induced DNA DSBs in NHEJ-deficient cells in the G2 cell cycle phase [72]. Instead, we observed a similarly reduced survival of Rad54^−/−^/LigIV^−/−^ MEFs and Rad54^−/−^ MEFs after proton irradiation and a better survival than LigIV^−/−^ MEFs. At present we cannot explain this phenomenon. We believe that the discrepancy observed in the response of the MEFs between photon and proton irradiation in the knockout strains is mainly due to the more pronounced sensitivity of Rad54^−/−^ cells to the cytotoxic effects to proton irradiation, which is also reflected by the higher levels of residual γH2A.X foci observed in Rad54^−/−^ cells only after proton irradiation. 

Interestingly, MRN-complex-dependent end resection of DNA DSBs is necessary for both HRR and alt-EJ [74,75]. One might thus speculate that in cells with a severe NHEJ defect a competition between HRR and alt-EJ repair might delay DNA DSBs repair, and thereby further contribute to the higher amounts of residual DNA damage foci, loss of genomic stability and higher radisensitivity of Lig IV^−/−^ cells. HRR is a cell cycle-dependent pathway, whereas alt-EJ operates regardless of the cell cycle. In cells with NHEJ deficiency, alt-EJ may become more prominent even if an end resection has occurred, so that additional HRR defects will not further compromise radiosensitivity at least upon X-ray photon irradiation [72,73]. Instead, the altered biology of the DNA damage observed upon proton irradiation may cause a higher dependency on HRR [28,29]. Herein, others revealed that clustered DNA damage induces chromatin destabilization resulting in exclusion of HRR as a possible DNA repair pathway and strongly increasing the contribution and importance of alt-EJ to DSB repair [76]. Following this, cells that failed in HRR used alt-EJ as a backup for DNA DSBs repair [77]. Yet, further mechanistic work is required to reveal if activation of alt-EJ as an adaptation process to inactivation of NHEJ or both major DSBs repair pathways [35] or other mechanisms contribute to the above findings. 

Instead, neither loss of XLF nor loss of PAXX had a significant impact on survival of fibroblasts after any type of irradiation, pointing to a redundant function of those NHEJ factors. We speculate that only the loss of key factors of the NHEJ complex, such as Lig IV, XRCC4 or DNA-PKcs, has a significant impact on clonogenic survival. However, XLF-deficiency affected the proper DNA repair kinetics after SOBP proton irradiation, pointing to its role in DNA repair and presumably in stabilizing the NHEJ-complex, but not in long-term survival. We speculate that loss of XLF can be compensated for and is therefore not essential for cells to survive.

To corroborate our findings on the impact of DNA repair-deficiencies on radiation response in cancer cell models, we used the DNA-PKcs-deficient M059J and DNA-PKcs-proficient M059K glioblastoma cell lines as well as the pancreatic cell lines BxPC3 with intact BRCA2 and Capan-1 with reported deficiency in BRCA2 expression. Similar to fibroblasts with impaired NHEJ due to loss of Lig IV, DNA-PKcs-deficient glioblastoma cells were more sensitive than DNA-PKcs-proficient M059K cells to irradiation with both X-ray photons and protons (EP and SOBP). However, calculated RBE values for 10% of clonogenic survival for EP and SOBP were approx. 1, which has already been observed by others [28,78]. These findings reveal that deficiency in NHEJ caused by the loss of DNA-PKcs already causes a dramatic radiosensitization to X-ray photon irradiation that overrides potential small differences in survival caused by the distinct biology of the DNA damage induced by X-ray photon and proton irradiation, respectively.

Though BRCA2-proficient BxPC3 and BRCA2-deficient Capan-1 pancreatic cancer cell lines were both more sensitive to irradiation with SOBP protons compared to irradiation with X-ray photons, the increase in the cytotoxic effects of proton irradiation was more pronounced in Capan-1 pancreatic cancer cells. However, BxPC3 and Capan-1 cells are not isogenic but constitute distinct cell lines and thus have a different genetic background [52,79]. Therefore, it is highly likely that the differences in the radiation response to photon and proton irradiation observed in BxPC3 and Capan-1 cells may be due to further, yet unknown, variations in other proteins or pathways involved in the regulation or execution of DNA DSB repair by NHEJ, HRR, or both [52,79]. Thus, mechanistic investigations with matched control cell lines, e.g., Capan-1 cells and Capan-1 cells with reconstituted BRCA2, or down-regulation of BRCA2 in HRR-proficient cancer cells, will be necessary to investigate functional relevance of BRCA2-deficiency for the observed radiosensitizing effect towards proton irradiation. 

Despite the severe impact of DNA-PKcs-deficiency on DNA repair kinetics in M059J cells upon proton and photon irradiation, X-ray photons had the highest impact on the number of the induction and removal of γH2A.X foci, as revealed by increased number of initial γH2A.X foci at 4 h and 8 h after irradiation. Moreover, deficiency in DNA-PKcs resulted in more pronounced damage persistence, as revealed by enhanced residual DNA damage foci in M059J cells 24 h upon irradiation with X-ray photons compared to SOBP protons. Our observations corroborate the major role of NHEJ for survival of irradiated cancer cells irrespective of the radiation quality, thereby confirming previous observations by others [46,78]. The importance of NHEJ in ensuring cell survival upon both proton and photon irradiation also corresponds to the reported involvement of NHEJ in the repair of approx. 80% of all DNA DSB occurring upon X-ray photon irradiation [46].

Taken together, while fibroblasts and cancer cells deficient in NHEJ (Lig IV^−/−^ or DNA-PKcs^−/−^) responded to proton and X-ray photon irradiation with a comparable reduction of clonogenic cell survival, the increase in residual DNA damage was higher after X-ray photon irradiation. We speculate that the repair of smaller DNA lesions induced by X-ray photons will mainly rely on NHEJ, so that cells with a deficiency in proper NHEJ will accumulate residual DNA damage [28,29]. In contrast, a delay in DNA repair kinetics of HRR-deficient cell lines was only observed after SOBP proton irradiation underlining the increased importance of HRR for the repair of clustered DNA damage induced by irradiation with SOBP protons [14,28,29,46,65,68,78]. However, the delay was less pronounced than the delay observed in NHEJ-deficient cells after X-ray photon irradiation. This suggests that NHEJ is also involved in repair of clustered DNA lesions induced by SOBP proton irradiation and that HRR plays an additional and supportive role in processing the induced DNA DSB, as suggested by others [46,78]. 

Generally, the cellular genetic make-up impacts radiosensitivity resulting in uncertainties in RBE, which are estimated to be 10–20% [19]. Furthermore, the tissue-dependent uncertainty in RBE is another limiting factor in tissue-related RBE estimation [80]. 

A better understanding of the characteristics and consequences of DNA damage induced by proton irradiation in normal tissues and tumors, and potential different molecular requirements for repair of DNA damage induced by proton irradiation is needed to harness the full potential of proton irradiation for clinical radiotherapy by combining proton radiotherapy with chemotherapy, or any other therapy in the future. Further joint efforts of the research community and properly consolidated basic research data are indispensable in order to define predictive markers allowing a patient stratification for proton radiotherapy based on molecular markers in the future. 

## 5. Conclusions

Super-resolution STED microscopy of wildtype fibroblast MEFs exposed to a similar physical dose of X-ray photons or SOBP protons revealed that SOBP protons induced increased numbers of clustered γH2A.X foci composed of several smaller foci. In contrast, irradiation with X-ray photons caused homogenously distributed small DNA damage foci. Furthermore, deficiency in key proteins involved in NHEJ, Lig IV and DNA-PKcs, delayed DNA repair after X-ray photon and proton irradiation compared to DNA repair-proficient control cells. Of note, deficiency in components of HRR compromised DNA repair and removal of γ-H2A.X foci particularly in response to SOBP proton irradiation. In line with these findings, NHEJ-deficiency resulted in significant sensitization of normal fibroblasts and cancer cells to both X-ray photons and protons, whereas deficiency in HRR proteins resulted in a specific sensitization to proton irradiation. We conclude that NHEJ plays an essential role in processing DNA DSB induced by proton and photon irradiation, whereas HRR gains increasing importance when proton irradiation is used, presumably for proper repair of the more clustered DNA lesions.

## Supplementary Materials

The following are available online at https://www.mdpi.com/2073-4409/9/4/889/s1, Figure S1: Clonogenic survival and DNA damage response of RPE-1 PAXX^−/−^ and 2BN hTERT XLF^−/−^ cells as well as respective control cells.

## References

[1] Begg A.C., Stewart F.A., Vens C. (2011). Strategies to improve radiotherapy with targeted drugs. Nat. Rev. Cancer.

[2] De Ruysscher D., Niedermann G., Burnet N.G., Siva S., Lee A.W.M., Hegi-Johnson F. (2019). Radiotherapy toxicity. Nat. Rev. Dis. Prim..

[3] Bristow R.G., Alexander B., Baumann M., Bratman S.V., Brown J.M., Camphausen K., Choyke P., Citrin D., Contessa J.N., Dicker A. (2018). Combining precision radiotherapy with molecular targeting and immunomodulatory agents: A guideline by the American Society for Radiation Oncology. Lancet Oncol..

[4] Orth M., Lauber K., Niyazi M., Friedl A.A., Li M., Maihöfer C., Schüttrumpf L., Ernst A., Niemöller O.M., Belka C. (2014). Current concepts in clinical radiation oncology. Radiat. Environ. Biophys..

[5] Baumann B.C., Mitra N., Harton J., Xiao Y., Wojcieszynski A., Gabriel P.E., Zhong H., Geng H., Doucette A., Wei J.J. (2019). Comparative effectiveness of proton therapy versus photon therapy as part of concurrent chemoradiotherapy for locally advanced cancer. J. Clin. Oncol..

[6] Durante M., Loeffler J.S. (2010). Charged particles in radiation oncology. Nat. Rev. Clin. Oncol..

[7] McDonald M.W., Zolali-Meybodi O., Lehnert S.J., Estabrook N.C., Liu Y., Cohen-Gadol A.A., Moore M.G. (2016). Reirradiation of Recurrent and Second Primary Head and Neck Cancer With Proton Therapy. Int. J. Radiat. Oncol..

[8] Rombi B., Vennarini S., Vinante L., Ravanelli D., Amichetti M. (2014). Proton radiotherapy for pediatric tumors: Review of first clinical results. Ital. J. Pediatr..

[9] Amichetti M., Cianchetti M., Amelio D., Enrici R.M., Minniti G. (2009). Proton therapy in chordoma of the base of the skull: A systematic review. Neurosurg. Rev..

[10] Tallen G., Resch A., Calaminus G., Wiener A., Leiss U., Pletschko T., Friedrich C., Langer T., Grabow D., Driever P.H. (2015). Strategies to improve the quality of survival for childhood brain tumour survivors. Eur. J. Paediatr. Neurol..

[11] Timmermann B. (2010). Proton Beam Therapy for Childhood Malignancies: Status Report. Klin. Pädiatrie.

[12] Tommasino F., Durante M. (2015). Proton radiobiology. Cancers.

[13] Jones B. (2017). Proton radiobiology and its clinical implications. Ecancermedicalscience.

[14] Oeck S., Szymonowicz K., Wiel G., Krysztofiak A., Lambert J., Koska B., Iliakis G., Timmermann B., Jendrossek V. (2018). Relating Linear Energy Transfer to the Formation and Resolution of DNA Repair Foci After Irradiation with Equal Doses of X-ray Photons, Plateau, or Bragg-Peak Protons. Int. J. Mol. Sci..

[15] Jermann M. (2015). Particle Therapy Statistics in 2014. Int. J. Part. Ther..

[16] Montay-Gruel P., Meziani L., Yakkala C., Vozenin M.C. (2019). Expanding the therapeutic index of radiation therapy by normal tissue protection. Br. J. Radiol..

[17] Nickoloff J.A. (2015). Photon, light ion, and heavy ion cancer radiotherapy: Paths from physics and biology to clinical practice. Ann. Transl. Med..

[18] Paganetti H. (2014). Relative biological effectiveness (RBE) values for proton beam therapy. Variations as a function of biological endpoint, dose, and linear energy transfer. Phys. Med. Biol..

[19] Paganetti H., Niemierko A., Ancukiewicz M., Gerweck L.E., Goitein M., Loeffler J.S., Suit H.D. (2002). Relative biological effectiveness (RBE) values for proton beam therapy. Int. J. Radiat. Oncol..

[20] Paganetti H., van Luijk P. (2013). Biological Considerations When Comparing Proton Therapy With Photon Therapy. Semin. Radiat. Oncol..

[21] Karger C.P., Peschke P. (2018). RBE and related modeling in carbon-ion therapy. Phys. Med. Biol..

[22] Willers H., Allen A., Grosshans D., McMahon S.J., von Neubeck C., Wiese C., Vikram B. (2018). Toward A variable RBE for proton beam therapy. Radiother. Oncol..

[23] Paganetti H., Olko P., Kobus H., Becker R., Schmitz T., Waligorski M.P.R., Filges D., Müller-Gärtner H.-W. (1997). Calculation of relative biological effectiveness for proton beams using biological weighting functions. Int. J. Radiat. Oncol..

[24] Scalliet P., Gueulette J. (2018). Radiobiological Characterization of Clinical Proton and Carbon-Ion Beams. arXiv.

[25] Held K.D., Kawamura H., Kaminuma T., Paz A.E.S., Yoshida Y., Liu Q., Willers H., Takahashi A. (2016). Effects of Charged Particles on Human Tumor Cells. Front. Oncol..

[26] Lühr A., von Neubeck C., Krause M., Troost E.G.C. (2018). Relative biological effectiveness in proton beam therapy – Current knowledge and future challenges. Clin. Transl. Radiat. Oncol..

[27] Suzuki M., Kase Y., Yamaguchi H., Kanai T., Ando K. (2000). Relative biological effectiveness for cell-killing effect on various human cell lines irradiated with heavy-ion medical accelerator in Chiba (HIMAC) carbon-ion beams. Int. J. Radiat. Oncol..

[28] Grosse N., Fontana A.O., Hug E.B., Lomax A., Coray A., Augsburger M., Paganetti H., Sartori A.A., Pruschy M. (2014). Deficiency in Homologous Recombination Renders Mammalian Cells More Sensitive to Proton Versus Photon Irradiation. Int. J. Radiat. Oncol..

[29] Fontana A.O., Augsburger M.A., Grosse N., Guckenberger M., Lomax A.J., Sartori A.A., Pruschy M.N. (2015). Differential DNA repair pathway choice in cancer cells after proton- and photon-irradiation. Radiother. Oncol..

[30] Liu Q., Ghosh P., Magpayo N., Testa M., Tang S., Gheorghiu L., Biggs P., Paganetti H., Efstathiou J.A., Lu H.-M. (2015). Lung Cancer Cell Line Screen Links Fanconi Anemia/BRCA Pathway Defects to Increased Relative Biological Effectiveness of Proton Radiation. Int. J. Radiat. Oncol..

[31] Peeler C.R., Mirkovic D., Titt U., Blanchard P., Gunther J.R., Mahajan A., Mohan R., Grosshans D.R. (2016). Clinical evidence of variable proton biological effectiveness in pediatric patients treated for ependymoma. Radiother. Oncol..

[32] Mladenov E., Magin S., Soni A., Iliakis G. (2013). DNA Double-Strand Break Repair as Determinant of Cellular Radiosensitivity to Killing and Target in Radiation Therapy. Front. Oncol..

[33] Roos W.P., Thomas A.D., Kaina B. (2015). DNA damage and the balance between survival and death in cancer biology. Nat. Rev. Cancer.

[34] Schipler A., Iliakis G. (2013). DNA double-strand-break complexity levels and their possible contributions to the probability for error-prone processing and repair pathway choice. Nucleic Acids Res..

[35] Iliakis G., Murmann T., Soni A. (2015). Alternative end-joining repair pathways are the ultimate backup for abrogated classical non-homologous end-joining and homologous recombination repair: Implications for the formation of chromosome translocations. Mutat. Res. Toxicol. Environ. Mutagen..

[36] Soni A., Siemann M., Pantelias G.E., Iliakis G. (2015). Marked contribution of alternative end-joining to chromosome-translocation-formation by stochastically induced DNA double-strand-breaks in G2-phase human cells. Mutat. Res. Toxicol. Environ. Mutagen..

[37] Critchlow S.E., Bowater R.P., Jackson S.P. (1997). Mammalian DNA double-strand break repair protein XRCC4 interacts with DNA ligase IV. Curr. Biol..

[38] Andres S.N., Modesti M., Tsai C.J., Chu G., Junop M.S. (2007). Crystal structure of human XLF: A twist in nonhomologous DNA end-joining. Mol. Cell.

[39] Davis A.J., Chen B.P.C., Chen D.J. (2014). DNA-PK: A dynamic enzyme in a versatile DSB repair pathway. DNA Repair (Amst)..

[40] Ochi T., Blackford A.N., Coates J., Jhujh S., Mehmood S., Tamura N., Travers J., Wu Q., Draviam V.M., Robinson C.V. (2015). DNA repair. PAXX, a paralog of XRCC4 and XLF, interacts with Ku to promote DNA double-strand break repair. Science.

[41] Heyer W.-D., Ehmsen K.T., Liu J. (2010). Regulation of Homologous Recombination in Eukaryotes. Annu. Rev. Genet..

[42] Lamarche B.J., Orazio N.I., Weitzman M.D. (2010). The MRN complex in double-strand break repair and telomere maintenance. FEBS Lett..

[43] Narod S.A., Salmena L. (2011). BRCA1 and BRCA2 mutations and breast cancer. Discov. Med..

[44] Chapman J.R., Taylor M.R.G., Boulton S.J. (2012). Playing the End Game: DNA Double-Strand Break Repair Pathway Choice. Mol. Cell.

[45] Bothmer A., Robbiani D.F., Di Virgilio M., Bunting S.F., Klein I.A., Feldhahn N., Barlow J., Chen H.-T., Bosque D., Callen E. (2011). Regulation of DNA end joining, resection, and immunoglobulin class switch recombination by 53BP1. Mol. Cell.

[46] Gerelchuluun A., Manabe E., Ishikawa T., Sun L., Itoh K., Sakae T., Suzuki K., Hirayama R., Asaithamby A., Chen D.J. (2015). The Major DNA Repair Pathway after Both Proton and Carbon-Ion Radiation is NHEJ, but the HR Pathway is More Relevant in Carbon Ions. Radiat. Res..

[47] Yang S.-H., Kuo T.-C., Wu H., Guo J.-C., Hsu C., Hsu C.-H., Tien Y.-W., Yeh K.-H., Cheng A.-L., Kuo S.-H. (2016). Perspectives on the combination of radiotherapy and targeted therapy with DNA repair inhibitors in the treatment of pancreatic cancer. World J. Gastroenterol..

[48] Biau J., Chautard E., Verrelle P., Dutreix M. (2019). Altering DNA Repair to Improve Radiation Therapy: Specific and Multiple Pathway Targeting. Front. Oncol..

[49] Bhattacharya S., Asaithamby A. (2017). Repurposing DNA repair factors to eradicate tumor cells upon radiotherapy. Transl. Cancer Res..

[50] Gavande N.S., VanderVere-Carozza P.S., Hinshaw H.D., Jalal S.I., Sears C.R., Pawelczak K.S., Turchi J.J. (2016). DNA repair targeted therapy: The past or future of cancer treatment?. Pharmacol. Ther..

[51] DiBiase S.J., Zeng Z.C., Chen R., Hyslop T., Curran W.J., Iliakis G. (2000). DNA-dependent protein kinase stimulates an independently active, nonhomologous, end-joining apparatus. Cancer Res..

[52] Abbott D.W., Holt J.T., Freeman M.L. (1998). Double-Strand Break Repair Deficiency and Radiation Sensitivity in BRCA2 Mutant Cancer Cells. JNCI J. Natl. Cancer Inst..

[53] Riballo E., Woodbine L., Stiff T., Walker S.A., Goodarzi A.A., Jeggo P.A. (2009). XLF-Cernunnos promotes DNA ligase IV–XRCC4 re-adenylation following ligation. Nucleic Acids Res..

[54] Franken N.A.P., Rodermond H.M., Stap J., Haveman J., van Bree C. (2006). Clonogenic assay of cells in vitro. Nat. Protoc..

[55] McMahon S.J. (2018). The linear quadratic model: Usage, interpretation and challenges. Phys. Med. Biol..

[56] Staab A., Zukowski D., Walenta S., Scholz M., Mueller-Klieser W. (2004). Response of Chinese Hamster V79 Multicellular Spheroids Exposed to High-Energy Carbon Ions. Radiat. Res..

[57] Oeck S., Malewicz N.M., Hurst S., Rudner J., Jendrossek V. (2015). The Focinator—A new open-source tool for high-throughput foci evaluation of DNA damage. Radiat. Oncol..

[58] Oeck S., Malewicz N.M., Hurst S., Al-Refae K., Krysztofiak A., Jendrossek V. (2017). The Focinator v2-0—Graphical Interface, Four Channels, Colocalization Analysis and Cell Phase Identification. Radiat. Res..

[59] Staudt T., Lang M.C., Medda R., Engelhardt J., Hell S.W. (2007). 2,2′-Thiodiethanol: A new water soluble mounting medium for high resolution optical microscopy. Microsc. Res. Tech..

[60] Baddeley A., Rubak E., Turner R. (2015). Spatial Point Patterns: Methodology and Applications with R.

[61] Olive P.L., Banáth J.P. (2006). The comet assay: A method to measure DNA damage in individual cells. Nat. Protoc..

[62] Gyori B.M., Venkatachalam G., Thiagarajan P.S., Hsu D., Clement M.-V. (2014). OpenComet: An automated tool for comet assay image analysis. Redox Biol..

[63] Krämer M., Weyrather W.K., Scholz M. (2003). The Increased Biological Effectiveness of Heavy Charged Particles: From Radiobiology to Treatment Planning. Technol. Cancer Res. Treat..

[64] Hojo H., Dohmae T., Hotta K., Kohno R., Motegi A., Yagishita A., Makinoshima H., Tsuchihara K., Akimoto T. (2017). Difference in the relative biological effectiveness and DNA damage repair processes in response to proton beam therapy according to the positions of the spread out Bragg peak. Radiat. Oncol..

[65] Reindl J., Girst S., Walsh D.W.M., Greubel C., Schwarz B., Siebenwirth C., Drexler G.A., Friedl A.A., Dollinger G. (2017). Chromatin organization revealed by nanostructure of irradiation induced γH2AX, 53BP1 and Rad51 foci. Sci. Rep..

[66] Chaudhary P., Marshall T.I., Currell F.J., Kacperek A., Schettino G., Prise K.M. (2016). Variations in the Processing of DNA Double-Strand Breaks Along 60-MeV Therapeutic Proton Beams. Int. J. Radiat. Oncol..

[67] Henthorn N.T., Warmenhoven J.W., Sotiropoulos M., Aitkenhead A.H., Smith E.A.K., Ingram S.P., Kirkby N.F., Chadwick A.L., Burnet N.G., Mackay R.I. (2019). Clinically relevant nanodosimetric simulation of DNA damage complexity from photons and protons. RSC Adv..

[68] Ray S., Cekanaviciute E., Lima I.P., Sørensen B.S., Costes S.V. (2018). Comparing Photon and Charged Particle Therapy Using DNA Damage Biomarkers. Int. J. Part. Ther..

[69] Maeda K., Yasui H., Yamamori T., Matsuura T., Takao S., Suzuki M., Matsuda A., Inanami O., Shirato H. (2016). A Nucleoside Anticancer Drug, 1-(3-C-Ethynyl-β-D-Ribo-Pentofuranosyl)Cytosine, Induces Depth-Dependent Enhancement of Tumor Cell Death in Spread-Out Bragg Peak (SOBP) of Proton Beam. PLoS ONE.

[70] Timm S., Lorat Y., Jakob B., Taucher-Scholz G., Rübe C.E. (2018). Clustered DNA damage concentrated in particle trajectories causes persistent large-scale rearrangements in chromatin architecture. Radiother. Oncol..

[71] Szabó E.R., Brand M., Hans S., Hideghéty K., Karsch L., Lessmann E., Pawelke J., Schürer M., Beyreuther E. (2018). Radiobiological effects and proton RBE determined by wildtype zebrafish embryos. PLoS ONE.

[72] Wu W., Wang M., Wu W., Singh S.K., Mussfeldt T., Iliakis G. (2008). Repair of radiation induced DNA double strand breaks by backup NHEJ is enhanced in G2. DNA Repair (Amst)..

[73] Iliakis G., Wang H., Perrault A.R., Boecker W., Rosidi B., Windhofer F., Wu W., Guan J., Terzoudi G., Pantelias G. (2004). Mechanisms of DNA double strand break repair and chromosome aberration formation. Cytogenet. Genome Res..

[74] Chang H.H.Y., Pannunzio N.R., Adachi N., Lieber M.R. (2017). Non-homologous DNA end joining and alternative pathways to double-strand break repair. Nat. Rev. Mol. Cell Biol..

[75] Daley J.M., Niu H., Miller A.S., Sung P. (2015). Biochemical mechanism of DSB end resection and its regulation. DNA Repair (Amst)..

[76] Iliakis G., Mladenova V., Sharif M., Chaudhary S., Mavragani I.V., Soni A., Saha J., Schipler A., Mladenov E. (2019). Defined Biological Models of High-Let Radiation Lesions. Radiat. Prot. Dosimetry.

[77] Howard S.M., Yanez D.A., Stark J.M. (2015). DNA Damage Response Factors from Diverse Pathways, Including DNA Crosslink Repair, Mediate Alternative End Joining. PLoS Genet..

[78] Bright S.J., Flint D.B., Chakraborty S., McFadden C.H., Yoon D.S., Bronk L., Titt U., Mohan R., Grosshans D.R., Sumazin P. (2019). Nonhomologous End Joining Is More Important Than Proton Linear Energy Transfer in Dictating Cell Death. Int. J. Radiat. Oncol. Biol. Phys..

[79] Deer E.L., González-Hernández J., Coursen J.D., Shea J.E., Ngatia J., Scaife C.L., Firpo M.A., Mulvihill S.J. (2010). Phenotype and genotype of pancreatic cancer cell lines. Pancreas.

[80] Paganetti H., Giantsoudi D. (2018). Relative Biological Effectiveness Uncertainties and Implications for Beam Arrangements and Dose Constraints in Proton Therapy. Semin. Radiat. Oncol..

