# Supplementary material for: Proton Irradiation Increases the Necessity for Homologous Recombination Repair Along with the Indispensability of Non-Homologous End Joining
Source: Cells. 2020 Apr 5;9(4):889. doi: 10.3390/cells9040889 (PMC7226794; doi:10.3390/cells9040889)
Supplement: Supplementary file 1 [file cells-09-00889-s001.pdf]

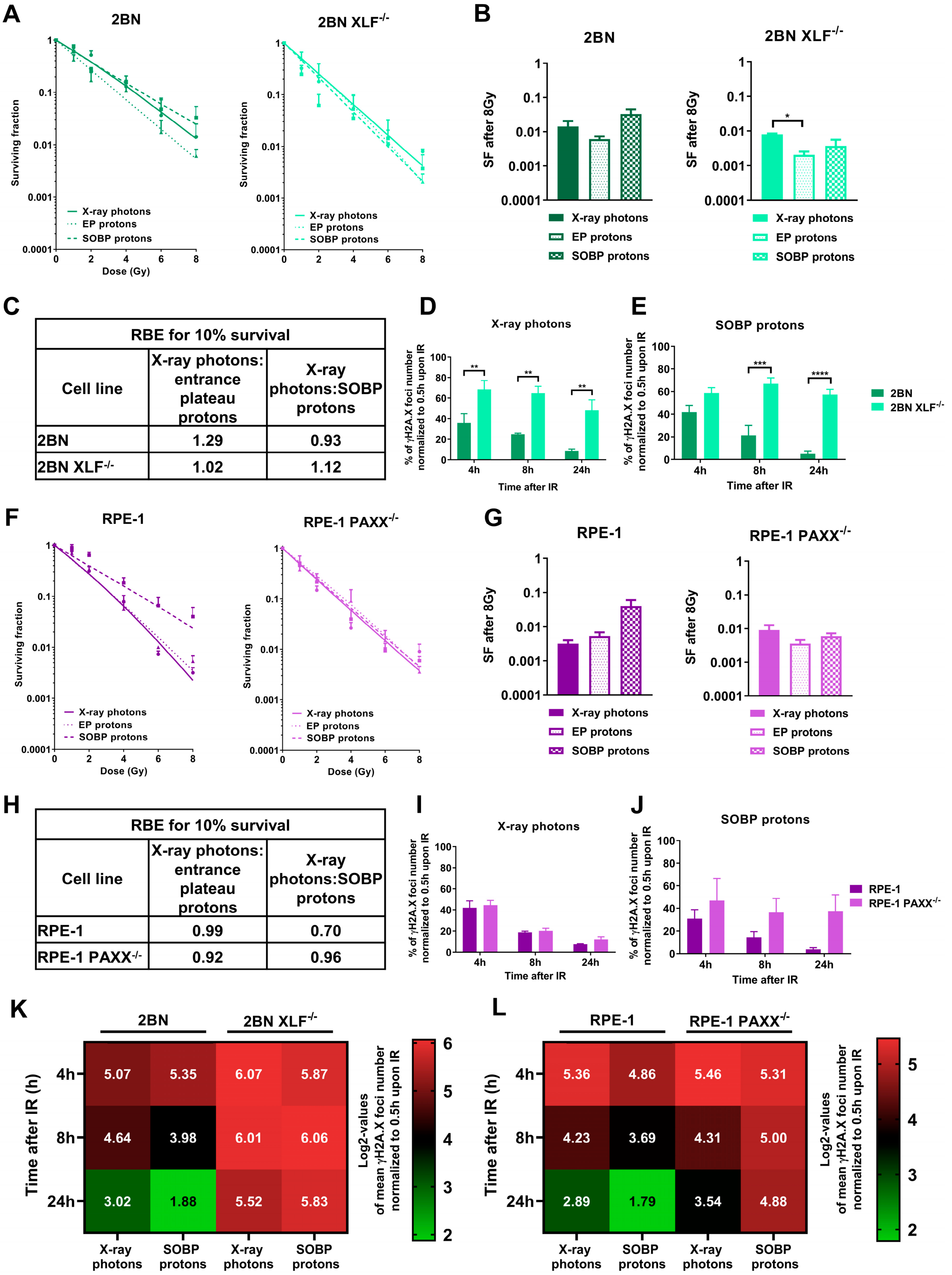

C

RBE for 10% survival		
Cell line	X-ray photons: entrance plateau protons	X-ray photons:SOBP protons
2BN	1.29	0.93
2BN XLF-/-	1.02	1.12

H

RBE for 10% survival		
Cell line	X-ray photons: entrance plateau protons	X-ray photons:SOBP protons
RPE-1	0.99	0.70
RPE-1 PAXX-/-	0.92	0.96

Figure S1. Clonogenic survival and DNA damage response of 2BN hTERT XLF−/− and RPE-1 PAXX−/− cells as well as respective control cells. (**A, B**) Clonogenic survival assay of 2BN hTERT XLF−/− cells irradiated with X-ray photons, EP and SOBP protons (1–8 Gy). (**C**) RBE values calculated for 10% survival of the indicated cell lines. (**D, E**) γH2A.X foci removal over time (4–24 h) normalized to initial foci number at 30 min in 2BN hTERT XLF−/− cells as well as respective control cells upon irradiation with 3 Gy of X-ray photons (**D**) and SOBP protons (**E**). (**F,G**) Clonogenic survival assay of RPE-1

PAXX$^{-/-}$ cells irradiated with X-ray photons, EP and SOBP protons (1–8 Gy). Data represent mean values ± SD from 3 independent experiments. One-way ANOVA. (**H**) RBE values calculated for 10% survival of the indicated cell lines. (**I, J**) γH2A.X foci removal over time (4–24 h) normalized to initial foci number at 30 min in RPE-1 PAXX$^{-/-}$ cells as well as respective control cells upon irradiation with 3 Gy of X-ray photons (**I**) and SOBP protons (**J**). (**K, L**) Comparison of radiation source effects in tested cell lines as log2 values of γH2A.X foci quantification. Data represent mean values ± SD (**A**,**F**) or SEM (**B**,**D**,**E**,**G**,**I**,**J**) from 3 independent experiments. One-way ANOVA with Tukey's multiple comparisons post-test (**B**,**G**) or two-way ANOVA with Tukey's multiple comparisons post-test (**D**,**E**,**I**,**J**); $^{*}p < 0.05$; $^{**}p < 0.01$; $^{***}p < 0.001$.
